# Native Valve Infective Endocarditis Secondary to Acute Cholecystitis in the Setting of Escherichia coli Bacteremia: A Case Report

**DOI:** 10.7759/cureus.37516

**Published:** 2023-04-13

**Authors:** Mara Jindeel, Gyuhee Seong

**Affiliations:** 1 School of Medicine, St. George’s University, West Indies, GRD; 2 Medicine, State University of New York (SUNY) Downstate Health Sciences University, Brooklyn, USA

**Keywords:** duke criteria, transesophageal echocardiography, hida cholescintigraphy, escherichia coli bacteremia, infective endocarditis

## Abstract

Infective endocarditis is an infection of the endocardium that affects the heart valves. It is usually caused by bacteremia secondary to distant infections such as urinary tract infections, surgical procedures, or other sources of pathogenic entry into the blood. It often affects damaged native valves, as well as prosthetic valves, and is primarily caused by Gram-positive bacteria, such as *Staphylococcus aureus*. Infective endocarditis secondary to *Escherichia coli* is rare, despite *E. coli *being one of the most common pathogens causing Gram-negative bacteremia. Between 1909 and 2002, 36 cases of native valve infective endocarditis were reported that met Duke criteria. The majority were secondary to urinary tract infections due to *E. coli*. Infective endocarditis secondary to *E. coli* bacteremia in the setting of acute cholecystitis is highly uncommon, and this case report aims to highlight this unusual presentation of infective endocarditis.

## Introduction

Infective endocarditis is an infection of the endocardium, especially affecting damaged or prosthetic heart valves. It is often caused by a secondary source of infection such as a distant source in the body, surgical sites, or nonsterile injections. Affected patients are generally older and often have underlying conditions, commonly diabetes mellitus [1], such as our patient. *Escherichia coli* endocarditis is a very rare condition, consisting of only 0.51% of cases of infective endocarditis [2]. Here we present a rare case of infective endocarditis in a native valve secondary to acute cholecystitis in the setting of *E. coli* bacteremia.

## Case presentation

**Figure 1:**
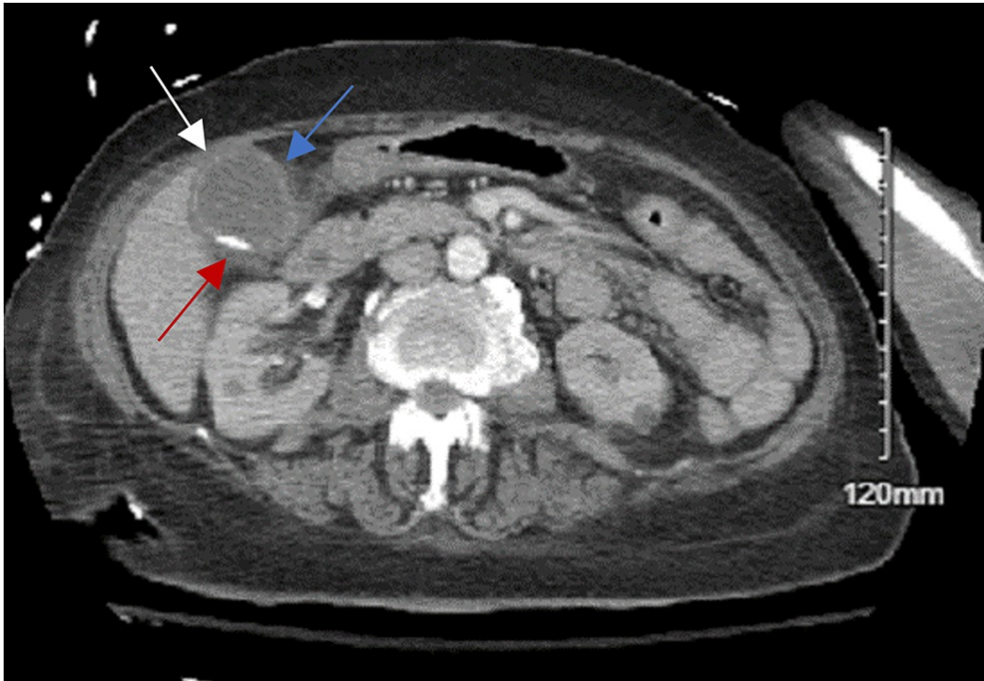
CT abdomen and pelvis with IV contrast. Distended gallbladder (white arrow), cholelithiasis (red arrow) with gallbladder wall thickening (blue arrow), concern for acute cholecystitis. No common bile duct dilatation.

Our patient is an 84-year-old female with a past medical history of hypertension, hyperlipidemia, diabetes mellitus, and dementia. She was initially admitted due to decreased appetite and diarrhea. She had fluid-responsive hypotension (80/50 mmHg) in the emergency department. Labs were significant for elevated white blood cell (WBC) to 13.99 K/uL. She also had hyponatremia, hyperkalemia, and acidosis with serum creatinine of 4.88 mg/dL. She had lactic acidosis to 15.9 mmol/L. Her liver function tests were also elevated. Urinalysis was positive for pyuria but negative for nitrites. CT head imaging did not demonstrate any evidence for acute intracranial hemorrhage or large territory infarction, but showed a meningioma (1.1 cm x 1.4 cm x 1.5 cm). CT scan of the abdomen and pelvis demonstrated cholelithiasis with pericholecystic fluid, indicating possible acute cholecystitis (Figure 1). 

She was admitted to the medical intensive care unit (ICU) for metabolic acidosis and hyperkalemia in the setting of acute kidney injury and started on continuous renal replacement therapy. She was started on an infusion of bicarbonate. She was started on piperacillin-tazobactam, however, her bacteremia did not resolve. Repeat urinalysis was negative for infection. Piperacillin-tazobactam was discontinued, and she was then started on ceftriaxone and metronidazole, as *E. coli* was preliminarily identified on the blood culture. Later the metronidazole was discontinued because speciation of blood culture indicated *E. coli* bacteremia sensitive to ceftriaxone.

**Figure 2:**
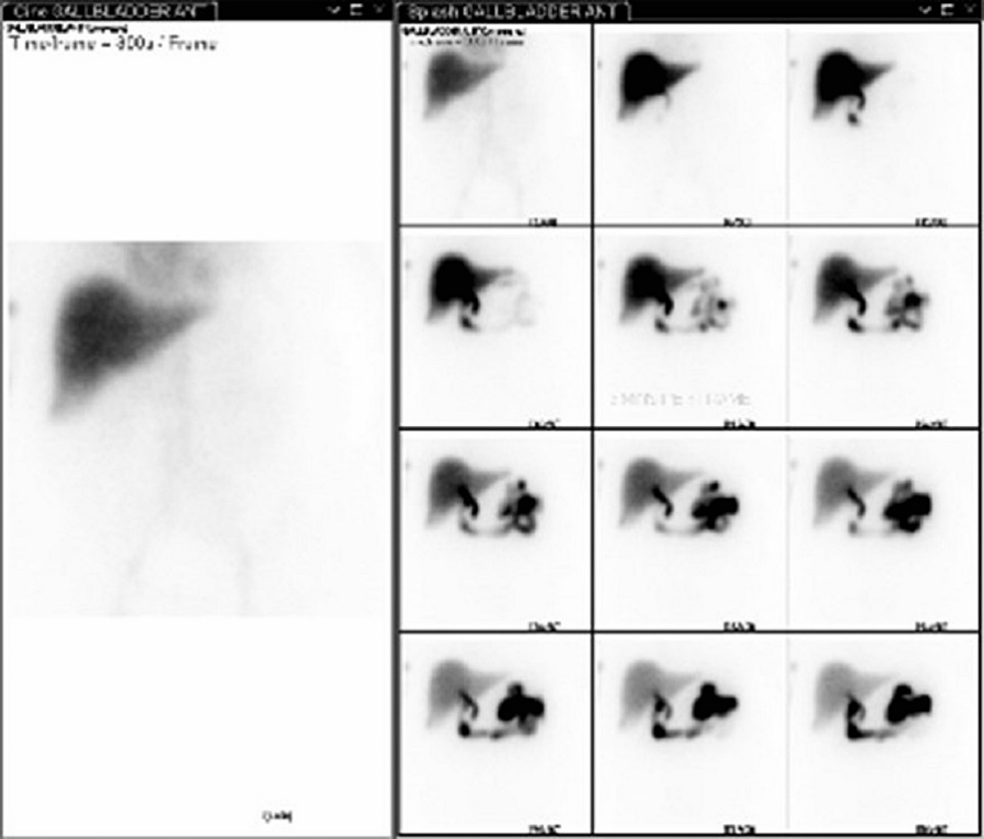
HIDA cholescintigraphy. The gallbladder is not visualized. This finding is consistent with acute cholecystitis in the appropriate clinical settings. HIDA, hepatobiliary iminodiacetic acid

**Figure 3:**
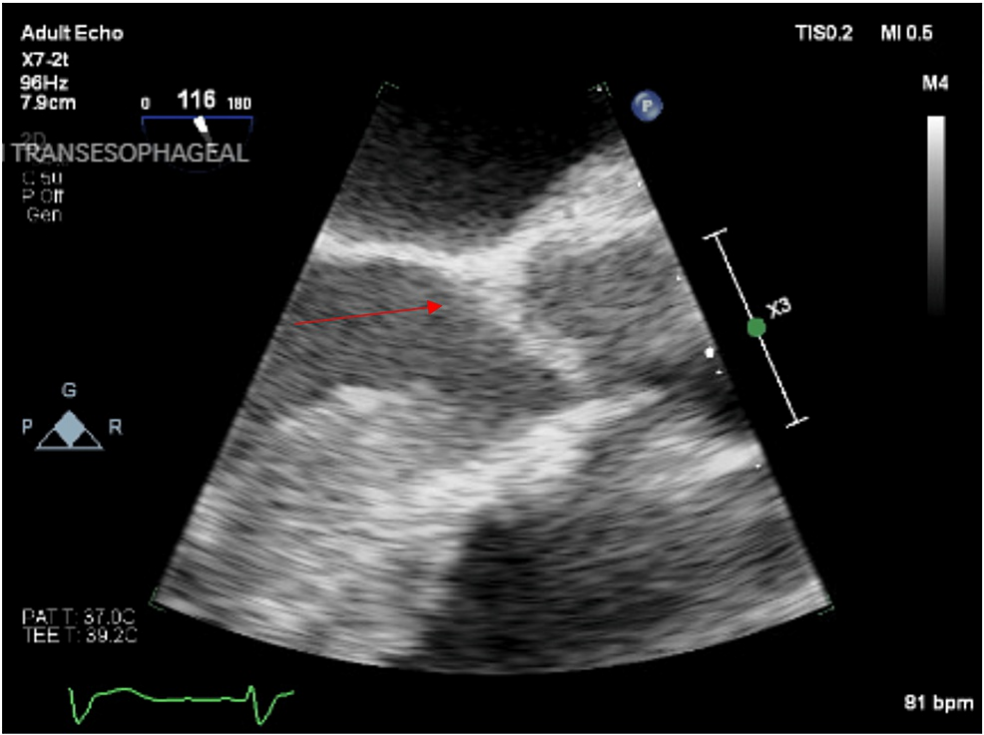
Transesophageal echocardiography. A small, loosely organized, mobile vegetation (red arrow) on the aortic valve

**Figure 4:**
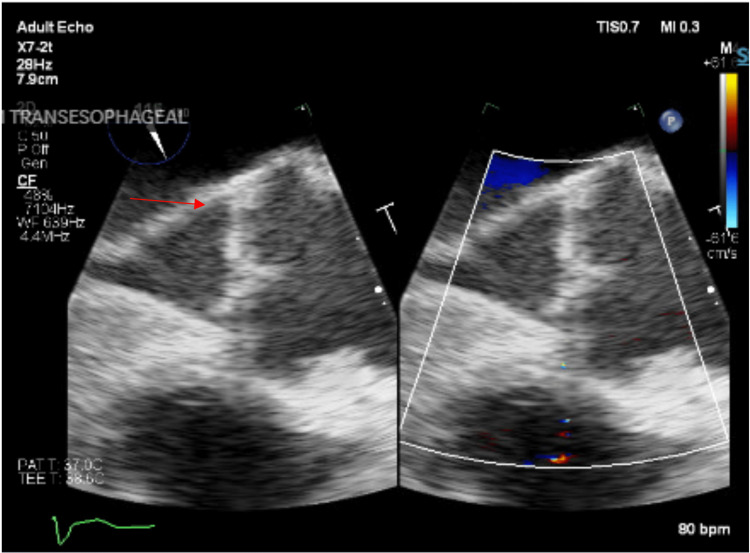
Transesophageal echocardiography with doppler. A small, loosely organized, mobile vegetation (red arrow) on the aortic valve, as characterized in Figure 3

A hepatobiliary iminodiacetic acid (HIDA) cholescintigraphy confirmed acute cholecystitis (Figure 2). A percutaneous transhepatic cholecystostomy tube was placed to drain the cholecystic fluid, and surgery was not performed because the patient was not a good surgical candidate. Persistent *E. coli* bacteremia in the setting of adequate antibiotics raised concern for cardiac seeding. Transesophageal echocardiography showed a small vegetation on the anterior surface (noncoronary cusp) of the aortic valve, indicative of infective endocarditis (Figure 3; see Figure 4 also). Subsequent blood cultures were negative for bacteremia. The patient continued on ceftriaxone and was discharged on a six-week course of daily oral levofloxacin with a percutaneous transhepatic cholecystostomy tube to remain in place for 6-8 weeks. 

## Discussion

Congenital or acquired native valves and prosthetic valves are the most commonly affected in infective endocarditis. In cases found in the literature, the majority affected the mitral valve [3]. In our case, the aortic valve was affected. Our patient had infective endocarditis due to *E. coli* bacteremia in the setting of acute cholecystitis, likely precipitated by cholelithiasis. She met the modified Duke criteria for a definitive diagnosis of infective endocarditis -- her blood cultures were positive for *E. coli* and transesophageal echocardiography showed cardiac vegetation. *E. coli* bacteremia, likely due to acute cholecystitis, seeded the anterior leaflet of the aortic valve. Although infective endocarditis is a rare disease, its rates are rising. Older women, especially those with diabetes, are disproportionately affected. 

Although *E. coli* is a rare cause of infective endocarditis, it is important to note that not all strains of this bacteria are equally pathogenic to the myocardium. Strains that are found outside of the gastrointestinal tract, termed extra-intestinal pathogenic *E. coli* (ExPEC) are more adept at colonizing cardiac tissue due to their specific virulence factors. These strains are mainly a part of the phylogenetic group B2 classification [4]. Another interesting note is that infective endocarditis due to *E. coli* has a higher mortality rate than endocarditis caused by the HACEK-group Gram-negative bacteria (Haemophilus spp., Aggregatibacter spp., Cardiobacterium hominis, Eikenella corrodens, and Kingela spp.) [5]. Despite the higher mortality rate of *E. coli* endocarditis, our patient recovered without surgical intervention [3].

As previously stated, infective endocarditis due to acute cholecystitis is extremely uncommon. After a literature review, we found only one case on Pubmed of *E. coli* endocarditis due to acute acalculous cholecystitis. The case referred to a 67-year-old male patient with mitral regurgitation, valve perforation, left ventricular pseudoaneurysm formation, congestive heart failure, spondylodiscitis, and endophthalmitis, which led to endocarditis of the posterior leaflet of the mitral valve. The mitral valve replacement for this patient was successful [6].

A review article by Branger et al. well demonstrated that over half of the infective endocarditis cases affect the mitral valve, with only 11 out of 37 cases affecting the aortic valve. In addition, renal insufficiency may be an associated factor since 12 out of 32 previously reported patients with *E. coli* endocarditis experienced renal insufficiency, like our case. Our patient had acute renal failure and initially required continuous renal replacement therapy in the ICU [3].

## Conclusions

Cases of infective endocarditis, especially those caused by *E. coli* bacteremia, are uncommon. Our case demonstrates that although infective endocarditis secondary to acute cholecystitis in the setting of *E. coli* bacteremia is a rare presentation of this disease, it can still occur, especially in certain populations. Those include older patients with other underlying health conditions, such as diabetes mellitus, and damaged native heart valves. *E. coli* endocarditis due to acute cholecystitis is very rare, with only a few reported so far. Patients in this demographic presenting with *E. coli* bacteremia may benefit from being screened for infective endocarditis.
